# Long live FOXO: unraveling the role of FOXO proteins in aging and longevity

**DOI:** 10.1111/acel.12427

**Published:** 2015-12-08

**Authors:** Rute Martins, Gordon J. Lithgow, Wolfgang Link

**Affiliations:** ^1^ Regenerative Medicine ProgramDepartment of Biomedical Sciences and MedicineUniversity of AlgarveCampus de Gambelas8005‐139FaroPortugal; ^2^ The Buck Institute for Research on AgingNovatoCA94945USA; ^3^ Centre for Biomedical Research (CBMR)University of AlgarveCampus de Gambelas8005‐139FaroPortugal

**Keywords:** aging, animal models, FOXO transcription factors, insulin and IGF‐1 signaling pathway, longevity

## Abstract

Aging constitutes the key risk factor for age‐related diseases such as cancer and cardiovascular and neurodegenerative disorders. Human longevity and healthy aging are complex phenotypes influenced by both environmental and genetic factors. The fact that genetic contribution to lifespan strongly increases with greater age provides basis for research on which “protective genes” are carried by long‐lived individuals. Studies have consistently revealed FOXO (Forkhead box O) transcription factors as important determinants in aging and longevity. FOXO proteins represent a subfamily of transcription factors conserved from *Caenorhabditis elegans* to mammals that act as key regulators of longevity downstream of insulin and insulin‐like growth factor signaling. Invertebrate genomes have one FOXO gene, while mammals have four FOXO genes: FOXO1, FOXO3, FOXO4, and FOXO6. In mammals, this subfamily is involved in a wide range of crucial cellular processes regulating stress resistance, metabolism, cell cycle arrest, and apoptosis. Their role in longevity determination is complex and remains to be fully elucidated. Throughout this review, the mechanisms by which FOXO factors contribute to longevity will be discussed in diverse animal models, from *Hydra* to mammals. Moreover, compelling evidence of FOXOs as contributors for extreme longevity and health span in humans will be addressed.

**Abbreviations**
4E‐BP: 4E‐binding protein
ADIPOQ: adiponectin gene (structural homology to complement factor C1q)
AGE‐1: aging alteration 1
AFX: acute leukemia fusion gene located in chromosome X
AKT: human homolog of viral oncogene v‐akt
AMP: adenosine monophosphate
AMPK: AMP‐activated protein kinase
APG: autophagy
APOE: apolipoprotein E
aqp‐1: aquaporin 1
ATP: adenosine triphosphate
AUT10: autophagocytosis 10
bec‐1: beclin 1
CHARGE: cohorts for heart and aging research in genomic epidemiology
CRM1: chromosome region maintenance 1
COQ7: coenzyme Q7 homolog
DAF: dauer formation
dFoxO: *Drosophila* FOXO
Dilps: *Drosophila* insulin‐like peptides
DNA: deoxyribonucleic acid
DR: dietary restriction
FIRKO: fat‐specific insulin receptor knockout
FKHR: Forkhead in rhabdomyosarcoma
FKHRL1: Forkhead in rhabdomyosarcoma like protein 1
FOXA: Forkhead box transcription factors of the class A
FOXO: Forkhead box transcription factors of the class O
FOXS: Forkhead box transcription factors of the class S
GFP: green fluorescent protein
GH: growth hormone
GWAS: genomewide association scan
HSCs: hematopoietic stem cells
HOMA: homeostasis model assessment
HSF‐1: heat‐shock factor 1
IGF‐1: insulin‐like growth factor 1
IGF1R: insulin‐like growth factor 1 receptor
IIS: insulin and IGF‐1 signaling pathway
InR: insulin‐like receptor
Ins: insulin‐like peptide
JNK: Jun N‐terminal kinase
KRI‐1: *Caenorhabditis elegans* orthologous to human Krev interaction trapped 1 (KRIT1)
L1‐L4: larval stages of *C. elegans*
MAF: minor allele frequency
mTOR: mammalian target of rapamycin
Nrf: nuclear respiratory factor
PDK‐1: 3‐phosphoinositide‐dependent kinase‐1
PHA‐4: pharynx 4‐cell embryo
PI3K: phosphoinositide 3‐kinase
PSMD11: proteasome (PSM), non‐ATPase subunit (D) 11
PTEN: phosphatase and tensin homolog
RNA: ribonucleic acid
S6K1: ribosomal S6 protein kinase 1
SCL‐1: SCP‐like extracellular protein 1
SGK: serum‐ and glucocorticoid‐inducible protein kinase
SIRT1: sirtuin 1
sir‐2.1: sirtuin 2.1
SKN‐1: skinhead 1
SNPs: single nucleotide polymorphisms
STACs: sirtuin‐activating compounds
TCER: transcription elongation regulator
TOR: target of rapamycin
VPS30: vacuolar protein sorting 30

## Introduction

Aging can be generally defined has the functional deterioration of physiological mechanisms which strictly depends on the passage of time. This decline constitutes the key risk factor for age‐related diseases such as cancer and cardiovascular and neurodegenerative disorders. It is therefore not surprising that humankind has put considerable efforts in understanding the processes of aging (and how to delay them) since ancient times.

Human longevity and healthy aging are complex phenotypes influenced by environmental (diet, physical activity, health habits, and psychosocial factors) and genetic factors (Herskind *et al*., 1996; Christensen *et al*., 2006; Bishop & Guarente, 2007). Heritability accounts for ≈25% of lifespan in an average‐lived population. The genetic contribution of lifespan increases with greater age, particularly after the age of 60, reaching estimates of 33% in women and 48% in men living to at least 100 (Sebastiani & Perls, 2012; Brooks‐Wilson, 2013). Accordingly, longevity clusters within families as parents and siblings of centenarians have an increased probability of reaching advanced age (Perls *et al*., 2000, 2002; Atzmon *et al*., 2004; Willcox *et al*., 2006).

It has become increasingly evident that lifespan is closely related to health span and that long‐lived individuals develop chronic illnesses (both physical and cognitive) later in life, thereby confirming the compression of morbidity hypothesis proposed by Fries in 1980 (Fries, 1980; Hitt *et al*., 1999; Andersen *et al*., 2012). A study performed in centenarians revealed that there is a progressive compression of disability and morbidity such that at the remarkable survival age of 110, subjects presented with age‐related diseases in the last 5.2% of their lives (vs. 17.9% in controls, 9.4% in individuals 100–104, and 8.9% in individuals 105–109) (Andersen *et al*., 2012). As extreme longevity appears to be a result of genetic factors more than environmental ones, when compared to an average lifespan, significant effort has gone into determining which genetic variants can slow aging and diminish the risk for age‐related diseases. Interestingly, the Leiden Longevity Study showed that, by GWAS, genomes of nonagenarians carry the same number of disease risk alleles for coronary artery disease, cancer and type 2 diabetes as young controls (Beekman *et al*., 2010). These results hint toward the perception that these long‐lived individuals could carry “protective genes” that may work in general cellular defense mechanisms, for example, against oxidative stress. In fact, several studies have consistently revealed APOE and FOXOs (FOXO1 and FOXO3) as “longevity genes” (Willcox *et al*., 2006; Anselmi *et al*., 2009; Flachsbart *et al*., 2009; Soerensen *et al*., 2010, 2015; Brooks‐Wilson, 2013; Bao *et al*., 2014; Broer *et al*., 2015). Aging has long been considered a process of degradation occurring in a random fashion that would lead to the accumulation of cellular damage in a stochastic fashion and, consequently, tissue decline and death. However, it is now known that aging can be modulated by genetic pathways and biochemical processes which are evolutionarily conserved (Kenyon, 2010b; Lopez‐Otin *et al*., 2013). According to the quasi‐programmed theory, aging is not programmed, but rather a consequence of genetic programs that determine developmental growth early in life (Blagosklonny, 2013a,b). Lopez‐Otin *et al*. (2013) in an attempt to define common denominators of aging in different organisms have defined nine cellular and molecular hallmarks of aging: genomic instability, telomere attrition, epigenetic alterations, loss of proteostasis, deregulated nutrient sensing, mitochondrial dysfunction, cellular senescence, stem cell exhaustion, and altered intercellular communication.

Among these hallmarks, the “deregulated nutrient sensing” was the first to be described to influence aging in animals, through the insulin and IGF‐1 signaling pathway (IIS) (Kenyon, 2005). IGF‐1 is produced by several cells types (mainly hepatocytes) in response to GH release from the anterior pituitary. IGF‐1 has been shown to trigger the same intracellular signaling pathways stimulated by insulin. The IIS pathway is the most evolutionarily conserved pathway of aging, shown to modulate lifespan in model organisms across a great evolutionary distance from *Caenorhabditis elegans* to mice (Kimura *et al*., 1997; Tatar *et al*., 2001; Fontana *et al*., 2010; Kenyon, 2010b; Mercken *et al*., 2013). Accordingly, genetic polymorphisms/mutations that cause loss of function of GH, IGF‐1 receptor, insulin receptor or its downstream factors, have been implicated in human longevity as in model organisms (Fontana *et al*., 2010; Kenyon, 2010b; Tazearslan *et al*., 2011; Barzilai *et al*., 2012; Milman *et al*., 2014). Dietary restriction is a well‐known environmental signal shown to expand lifespan in eukaryote species, from yeast to primates (Colman *et al*., 2009; Fontana *et al*., 2010; Mattison *et al*., 2012). The “longevity response” to dietary restriction is regulated by several nutrient‐sensing pathways: the kinase TOR, AMP kinase, sirtuins, and the IIS (Kenyon, 2005).

## FOXO transcription factors

FOXO proteins are the most important transcriptional effectors of the IIS (Kenyon *et al*., 1993; Gottlieb & Ruvkun, 1994; Brunet *et al*., 1999; Dong *et al*., 2008). FOXOs represent a subfamily of the Forkhead family of transcription factors. This family is characterized by a conserved DNA‐binding domain (the Forkhead box or FOX) and comprises more than 100 members in humans, from FOXA to FOXS (Zanella *et al*., 2010; Genin *et al*., 2014). The FOXO subfamily is conserved from *C. elegans* to mammals but, while invertebrates have only one FOXO gene, mammals have four FOXO genes: FOXO1 (FKHR), FOXO3 (FKHRL1), FOXO4 (AFX), and FOXO6 (Kaestner *et al*., 2000; Hannenhalli & Kaestner, 2009). In mammals, this subfamily is involved in a wide range of crucial cellular processes regulating stress resistance, metabolism, cell cycle arrest, and apoptosis, but their role in longevity still remains to be elucidated. FOXO proteins function mainly as transcriptional activators by binding the consensus core recognition motif TTGTTTAC, and their activity is inhibited by the IIS pathway (Biggs *et al*., 1999; Brunet *et al*., 1999; Henderson & Johnson, 2001; Lin *et al*., 2001; Calnan & Brunet, 2008; Zanella *et al*., 2010; Webb & Brunet, 2014). Briefly, insulin or IGF‐1 triggers an intracellular pathway mediated by PI3K‐AKT, allowing phosphorylation of FOXO factors by the serine/threonine kinase AKT at three conserved residues within the FOXO proteins. AKT‐mediated phosphorylation of FOXO leads to its nuclear exclusion and, in turn, to suppression of FOXO‐dependent transcription of target genes (Guo *et al*., 1999; Murphy *et al*., 2003). Conversely, in the absence of growth factor signaling or upon cellular stress, FOXOs translocate into the nucleus and activate FOXO‐dependent gene expression. A diverse set of posttranslational modifications in addition to phosphorylation, such as acetylation/deacetylation, methylation, or ubiquitination has been shown to promote changes of subcellular localization, protein levels, DNA binding, and transcriptional activity of FOXO factors (Calnan & Brunet, 2008; Webb & Brunet, 2014) The combinatorial result of FOXO posttranslational modifications has been proposed to lead to the recruitment of specific FOXO‐binding partners regulating different FOXO‐dependent gene expression programs (Greer *et al*., 2007b; Calnan & Brunet, 2008; Hill *et al*., 2014). Several mechanisms of how FOXO proteins promote longevity have been suggested.

## FOXO and autophagy

Webb & Brunet (2014) have recently unveiled the role of FOXOs as prolongevity factors through the maintenance of protein homeostasis (proteostasis) (Morley *et al*., 2002; Hsu *et al*., 2003). In fact, it has been shown that FOXO factors participate in the regulation of genes responsible for two main mechanisms of intracellular clearance: autophagy and the ubiquitin‐proteasome system (Webb & Brunet, 2014). Defects in autophagy, the process of degradation and recycling of cytoplasmic proteins and organelles in response to starvation have been associated with premature aging and age‐related disorders (Hara *et al*., 2006; Komatsu *et al*., 2006; Jung *et al*., 2008; Pickford *et al*., 2008; Masiero *et al*., 2009; Lee *et al*., 2010a, 2010b). FOXOs affect the expression of genes involved in autophagy and mitophagy (muscle‐specific autophagy) in muscle cells from flies (dFOXO) to mammals (FOXO3), allowing adaptation of the tissues to starvation (Zhao *et al*., 2007; Sengupta *et al*., 2009; Demontis & Perrimon, 2010). Additionally, FOXO1 and FOXO3 activate autophagy mechanisms in diverse cell types: neurons, cardiomyocytes, renal tubular cells, and HSCs (Webb & Brunet, 2014). As mentioned, FOXOs are involved in the proteasome system degradation of short‐lived and regulatory cytosolic proteins. Aging is associated with a decreased proteasomal activity, leading to excess of damaged proteins in muscle, liver, and heart (Conconi *et al*., 1996; Petropoulos *et al*., 2000; Bulteau *et al*., 2002; Husom *et al*., 2004). Moreover, pathogenesis of neurodegenerative disorders such as Parkinson's, Alzheimer's, or Huntington's disease is generally related to an abnormal ubiquitin‐proteasome mechanism as either a primary cause or secondary consequence (Ciechanover & Brundin, 2003; Kikis *et al*., 2010; Webb & Brunet, 2014). FOXOs act on both the upregulation of ubiquitin ligases and by controlling the composition of the proteasome (Bodine *et al*., 2001; Sandri *et al*., 2004, 2006; Stitt *et al*., 2004; Vilchez *et al*., 2012). However, the direct effect of FOXO‐mediated proteostasis in mammals remains to be understood.

## FOXO and resistance to oxidative stress

One of the most significant functions of FOXO proteins is their role in cellular responses to oxidative stress. As accumulation of damage caused by ROS (reactive oxygen species) was postulated to be causative for aging, it has been hypothesized that FOXO factors influence aging and age‐related diseases by increasing the antioxidant capacity of cells (Kops *et al*., 2002; Storz, 2011). ROS play an important role as second messengers of cellular signaling and can lead to oxidative stress when cellular detoxification activity is decreased. As high and very low levels of ROS lead to impaired cellular functions, maintaining intracellular ROS homeostasis is essential to prevent pathological processes including cancer and other age‐associated diseases. FOXOs are regulated by oxidative stress via changes in upstream FOXO regulatory pathways or directly sensing the cellular redox status through reversible oxidation and reduction of cystein residues (Essers *et al*., 2004; Eijkelenboom & Burgering, 2013; Putker *et al*., 2013). FOXO factors regulate the expression of the key detoxification enzymes MnSOD (manganese superoxide dismutase), catalase, and GADD45 (Kops *et al*., 2002; Nemoto & Finkel, 2002). Accordingly, inactivation of Foxo factors has been shown to lead to intracellular accumulation of ROS promoting accelerated atherosclerosis, proliferation of transformed cells, and compromising long‐term proliferative potential of normal stem cells (Tothova *et al*., 2007; Tsuchiya *et al*., 2013).

## FOXO and stem cells

There is accumulating evidence that FOXO factors play an important role in stem cell biology and tissue homeostasis. During aging, the balance of removal and regeneration of cells in tissues becomes disturbed mainly due to a decrease in the regenerative potential of adult stem cells. Conditional deletion of Foxo1/3a/4 in the adult hematopoietic system of mice leads to apoptosis of HSCs preventing the repopulation of these stem cell populations. Similarly, aged mice in which Foxo3a was deleted display reduced regenerative potential (Miyamoto *et al*., 2007). Foxo‐deficient HSCs in these animals are thought to be driven out of quiescence into cell cycle, resulting in depletion of the stem cell pool (Tothova & Gilliland, 2007). Interestingly, the treatment of Foxo‐deficient mice with the antioxidant N‐acetylcysteine restores the HSC compartment, suggesting that the accumulation of ROS disturbs stem cell function. This observation is in line with the idea that decreased function of adult stem cells involved in the onset of age‐related diseases is secondary to the accumulation of cellular stress (Boyette & Tuan, 2014). The role of FOXO proteins in stem cell biology is not limited to adult stem cells. FOXO1 has been shown to directly control the expression of OCT4 and SOX2 two transcription factors critically involved in stemness. Accordingly, FOXO1 is necessary to maintain pluripotency of human ESC and the ortholog FOXO1 exerts a similar function in mouse ESCs (Zhang *et al*., 2011a).

**Figure 1:**
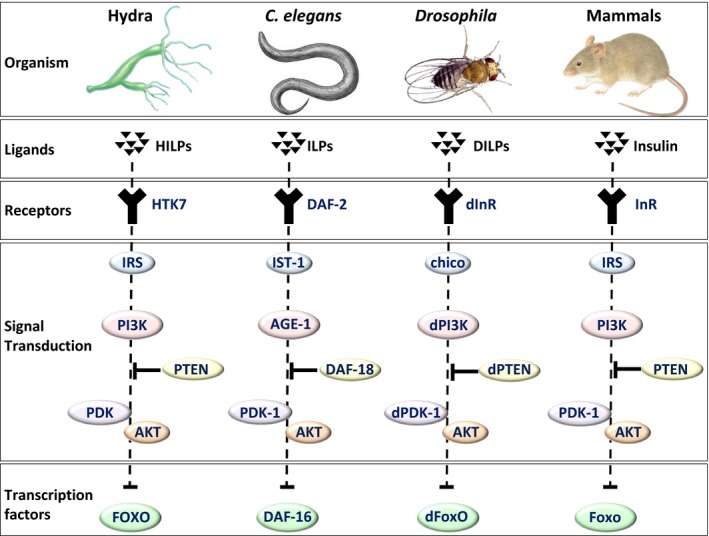
The key components of the PI3K signal transduction pathway are well conserved throughout evolution. The corresponding orthologues for these components in Hydra, Caenorhabditis elegans, Drosophila melanogaster and mammals are illustrated. It is important to note, that there is a single gene for many components, in Hydra, C. elegans and D. melanogaster whereas mammals have several isoforms of these components with the exception of PTEN. AGE‐1, ageing alteration 1; AKT, v‐akt murine thymoma viral oncogene homologue 1; DAF‐2, abnormal dauer formation‐2; FOXO, forkhead family of transcription factor; INR, insulin receptor; IRS, substrate; IST‐1, insulin receptor substrate (IRS)‐like adaptor; PDK, phosphatidylinositide‐dependent protein kinase 1; PTEN; phosphatase and tensin homologue.

In this review, we give an overview of the current evidence that implicates FOXO transcription factors in human longevity. Although the precise mechanism by which FOXO factors influence human aging is not understood, an overwhelming amount of data from several animal models including *Hydra vulgaris*,* C. elegans*,* Drosophila melanogaster*, and mice shed light on the critical functions of FOXO protein family members in aging. We discuss the FOXO activities which might be relevant for human longevity throughout this review organized by species (Fig. 1).

## Population studies

The understanding that the genetic contribution to lifespan strongly increases with greater age provides basis for research on which genetic differences distinguish centenarians from average‐lived individuals (Herskind *et al*., 1996; Hjelmborg *et al*., 2006; Murabito *et al*., 2012; Sebastiani & Perls, 2012; Brooks‐Wilson, 2013). Accordingly, longevity clusters within families as parents and siblings of centenarians present an increased probability of reaching advanced age (Perls *et al*., 2000, 2002; Atzmon *et al*., 2004; Willcox *et al*., 2006). Alleles which are enriched in centenarians most likely represent genes that are significant for longevity and therefore a significant number of studies have been done in the quest for these alleles/genes.

Willcox *et al*. (2008) first reported that genetic variation in FOXO3A was strongly associated with human longevity. In a long‐lived population of male Americans of Japanese ancestry (mean attained age = 97.9 vs. control group = 78.5), a nested‐case–control study of five candidate genes (ADIPOQ, FOXO1A, FOXO3A, SIRT1, and COQ7) was performed. Among these, only the rs2764264, rs13217795, and rs2802292 FOXO3A SNPs stood out as they were found to be associated with longevity and healthy aging (Willcox *et al*., 2008). These long‐lived men had lower prevalence of cancer and cardiovascular disease better self‐reported health as well as high physical and cognitive function, even though they were in average 11 years older than controls. Homozygosity for the G allele of FOXO3A rs2802292 was shown to confer a significant protection considering the prevalence of congestive heart disease. The same allele was associated with markedly lower insulin, log insulin, and HOMA (homeostasis model assessment) score in the control group (Willcox *et al*., 2008).

Anselmi *et al*. (2009) validated the association of these FOXO3 polymorphisms with extreme longevity in males from the Southern Italian Centenarian Study. In particular, rs2802288, a proxy of rs2802292, showed the best allelic association‐MAF (minor allele frequency). These results were confirmed in the German population (Flachsbart *et al*., 2009). An extensive collection of 1762 German centenarians/nonagenarians and younger controls evidenced FOXO3A polymorphisms with the ability to reach exceptional old age. This association was substantially stronger in centenarians than in nonagenarians, highlighting the importance of centenarians in genetic longevity research (Flachsbart *et al*., 2009). Furthermore, the variation in FOXO3A was replicated by both case–control and longitudinal data in Danish population (the oldest‐old vs. middle‐aged individuals) (Soerensen *et al*., 2010). Among the fifteen SNPs analyzed, they found association of eight SNPs with longevity: four previously reported (rs13217795, rs2764264, rs479744, and rs9400239) and four novel SNPs (rs12206094, rs13220810, rs7762395, and rs9486902) (Soerensen *et al*., 2010).

Li *et al*. (2009) analyzed six SNPs from FOXO1A and FOXO3A genes by comparing 761 centenarians and 1056 younger individuals (control group) of the Han Chinese population. They found two SNPs of FOXO1A to be negatively associated with longevity in women, rs2755209 and rs2755213. On the other hand, all three SNPs studied for FOXO3A (rs2253310, rs2802292, and rs4946936) were positively associated with longevity in both genders (Li *et al*., 2009). They have concluded that as FOXO1A is more strictly associated with human female longevity, the genetic contribution to longevity trait may be affected by genders (Li *et al*., 2009). Also in the Han Chinese population, Zeng *et al*. analyzed the independent and joint effects of the FOXO1A and FOXO3A SNPs on long‐term survival. These authors found substantial gender differences in the independent effects and showed that the positive effects of FOXO3A and negative effects of FOXO1A largely compensate each other, although FOXO3A has a stronger impact (Zeng *et al*., 2010). Others have also revealed the importance of genetic variations in the IIS pathway in long‐lived individuals from Italian, Japanese, Ashkenazi Jewish, or Dutch ethnicity (Arai *et al*., 2001; Bonafe *et al*., 2003; Kojima *et al*., 2004; Kuningas *et al*., 2007; Suh *et al*., 2008; Pawlikowska *et al*., 2009). A meta‐analysis comprising 11 independent case–control studies and 5241 cases from different ethnic groups revealed five FOXO3A polymorphisms as associated with longevity: rs2802292, rs2764264, rs13217795, rs1935949, and rs2802288 (Bao *et al*., 2014). Moreover, rs2802292 and rs2764264 polymorphism are male‐specific longevity polymorphisms that may potentially identify long‐lived men. FOXO3A along with APOE has consistently been associated with longevity in multiple independent studies (Wheeler & Kim, 2011; Brooks‐Wilson, 2013). Accordingly, Broer *et al*. (2015) conducted a meta‐analysis of GWAS with 6036 longevity cases (age ≥ 90 years) and 3757 controls (CHARGE consortium). Among the ~2.5 million SNPs analyzed, only the APOE and FOXO3 variants confirmed a significant association with longevity (Broer *et al*., 2015). Recent work by Soerensen *et al*. (2015) compared aging‐related traits (cognitive function, hand grip strength, activity of daily living, and self‐rated health) with 15 FOXO3A SNPs in Danish oldest‐old individuals. Gene‐based testing revealed a significant increase in activity of daily living and reduced bone fracture risk for carriers of the minor alleles of 8 and 10 FOXO3A SNPs, respectively (Soerensen *et al*., 2015). In summary, gathered evidence from Japanese Americans, Han Chinese, Californians, New Englanders, Ashkenazi Jews, Danish, Germans, and Italians shows that FOXO3A SNPs are associated with exceptional longevity. Although there is compelling evidence that FOXO3 gene sequence variants influence longevity, it remains to be determined how these variations translate into phenotypic characteristics that enable a long lifespan. FOXO3A alleles associated with longevity are intronic and not linked to known coding SNPs (Donlon *et al*., 2012; Murabito *et al*., 2012; Brooks‐Wilson, 2013), suggesting that these SNPs will most likely affect FOXO3A expression rather than protein activity.

## Animal models

### *Hydra vulgaris*

*Hydra vulgaris* is a freshwater radial‐symmetric polyp of the phylum *Cnidaria*, placed at the basal root of the animal life. Bridge *et al*. (2010) first described the presence of a single FoxO gene in *Hydra*. These authors found significant parallels in the regulation of FoxO between *Hydra* and bilaterian animals, showing that FoxO transcriptional activity is negatively modulated by the PI3K/AKT/SGK pathway and, accordingly, nuclear localization of a FoxO‐GFP fusion protein is significantly increased by a PI3K inhibitor (Bridge *et al*., 2010). Lasi *et al*. (2010) showed that transient expression of FoxO‐GFP protein induced an apoptosis rate of 20–60% in *Hydra* epithelial cells. Conversely, co‐expression of one of the *Hydra* insulin‐like genes with the FoxO‐GFP protein was shown to decrease the rate of apoptosis in these cells. As the IIS acts through the PI3K/AKT/SGK pathway, one can hypothesize that the IIS reduces *Hydra*′s FoxO activity (Lasi *et al*., 2010).

*Hydra* presents itself as unique model to study longevity due to its extraordinary regenerative abilities through the self‐renewal and differentiating capacities of its epithelial and interstitial stem cells. These properties are thought to be related to the fact that these animals routinely reproduce asexually (Wittlieb *et al*., 2006; Khalturin *et al*., 2007; Bosch, 2009; Bellantuono *et al*., 2015). Three stem cell lineages allow this continuous self‐renewal: ectodermal and endodermal epitheliomuscular stem cells and interstitial stem cells (Bosch, 2009; Bosch *et al*., 2010). The search for transcriptome‐specific signatures enabling the regulation of self‐renewal and differentiation showed that FoxO is highly expressed in the three stem cell lineages of *Hydra* (Boehm *et al*., 2012). The overexpression of FoxO in the multipotent interstitial stem cell lineages increases stem cell and progenitor cell proliferation and activates expression of stem cell genes in terminally differentiated somatic cells (Boehm *et al*., 2012). Conversely, silencing FoxO in epithelial cells increased the number of terminally differentiated cells (Boehm *et al*., 2012) Altogether, these results suggest a key role for FoxO in *Hydra*'s apparent biological immortality, specifically affecting its continuous self‐renewal capacity (Bellantuono *et al*., 2015). In a recent essay, Schaible and Sussman hypothesized that whereas FoxO is exclusively devoted to life‐prolonging cell renewal in *Hydra*, the evolutionary diversification of FoxO functions in multicellular eukaryotes that manifest aging resulted in a dilution of FoxO's rejuvenating capacity (Schaible & Sussman, 2013).

### *Caenorhabditis elegans*

*Caenorhabditis elegans* is a free‐living unsegmented pseudocoelomate member of the phylum Nematoda that has an average lifespan of about 2–3 weeks. It has two sexes: a self‐fertilizing hermaphrodite and a male. The life cycle of *C. elegans* is comprised of the embryonic stage, four larval stages (L1–L4), and adulthood. If the environmental conditions are not favorable (absence of food, high temperature, or presence of a pheromone indicating high population density), L1 worms develop into alternative larval form called the dauer larva (Fielenbach & Antebi, 2008). The role of the IIS pathway in aging was first discovered in *C. elegans* through mutations that extended lifespan. Mutations in DAF‐2 (dauer larvae formation‐2), a hormone receptor similar to insulin and IGF‐1 receptors, doubled the lifespan of the worm (Kenyon *et al*., 1993; Kimura *et al*., 1997). This lifespan extension caused by DAF‐2 mutations required the activity of DAF‐16, which encodes the single FoxO homolog in *C. elegans* (Kenyon *et al*., 1993; Lin *et al*., 1997; Ogg *et al*., 1997). DAF‐16 is inactivated via its nuclear export by the evolutionary conserved signaling pathway downstream of DAF‐2 receptor (Henderson & Johnson, 2001; Lee *et al*., 2001; Lin *et al*., 2001). HSF‐1, the *C. elegans* heat‐shock transcription factor, is also required in DAF‐2 mutants to extend lifespan. HSF‐1, along with DAF‐16, promotes longevity by activating specific longevity genes, including genes that encode small heat‐shock proteins (Garigan *et al*., 2002; Hsu *et al*., 2003; Walker & Lithgow, 2003; Morley & Morimoto, 2004). The importance of DAF‐18/PTEN tumor suppressor in longevity and dauer larva formation, through the regulation of the IIS pathway, has been established in the late 1990s by several research groups (Ogg & Ruvkun, 1998; Gil *et al*., 1999; Mihaylova *et al*., 1999; Rouault *et al*., 1999). DAF‐18/PTEN has been shown to regulate L1 arrest in the germ line, and insulin‐like signaling appears to be transduced by AGE‐1/PI3K during L1 arrest (Weinkove *et al*., 2006; Zhang *et al*., 2011b).

DAF‐16 functions as the major target of the IIS pathway (Kenyon *et al*., 1993; Gottlieb & Ruvkun, 1994). DAF‐16/FoxO has been shown to regulate hundreds of genes in *C. elegans* including those related to stress response, antimicrobial activity, and metabolism, unveiling DAF‐16/FoxO as a central player of a complex network involving multiple upstream pathways and downstream target genes (Lee *et al*., 2003; McElwee *et al*., 2003; Murphy *et al*., 2003). The lifespan expansion effect accomplished by DAF‐16/FOXO is most likely due to two isoform variants DAF‐16a and DAF‐16d/f (Kwon *et al*., 2010; Chen *et al*., 2015). Diverse approaches including different dietary regimens or using compounds that mimic dietary restriction have been employed to investigate the effect of dietary restriction on lifespan extension in *C. elegans* (Kenyon, 2010b). Chronic food limitation increases lifespan by downregulating TOR activity, which increases autophagy and diminishes translation, probably through PHA‐4/FOXA transcription factor and S6 kinase, respectively, in a DAF‐16/FoxO‐independent manner (Kaeberlein *et al*., 2005; Hansen *et al*., 2007; Pan *et al*., 2007; Sheaffer *et al*., 2008). AMPK (AMP kinase), an energy sensor for cellular AMP/ATP ratio that enables catabolic reactions for energy gain, has been shown to respond to dietary restriction by increasing stress resistance and extending longevity in *C. elegans* in a DAF‐16/FOXO‐dependent manner (Apfeld *et al*., 2004; Greer *et al*., 2007a). AMPK directly phosphorylates and activates DAF‐16/FoxO (Greer *et al*., 2007a). Dietary restriction with every‐other‐day feeding is likely to promote *C. elegans* longevity via downregulation of the IIS pathway (Honjoh *et al*., 2009). In *C. elegans,* overexpression of sirtuin gene *sir‐2.1* activates DAF‐16/FoxO by interacting with 14‐3‐3 proteins (Berdichevsky *et al*., 2006). The ability of sirtuins to directly deacetylate DAF‐16/FoxO together with the fact that IIS pathway mutants do not need *sir‐2.1* to promote longevity, suggests that sirtuins act on DAF‐16/FoxO independently of the IIS pathway (Kenyon, 2010b). Interestingly, whereas high levels of ROS led to premature death, low concentrations caused a prolongation of lifespan and this extension is dependent on both *daf‐16* and *sir‐2.1* genes (Heidler *et al*., 2010). Kim *et al*. (2014) have recently shown that antioxidant treatment can extend the lifespan of *C. elegans* through the phosphorylation and cytoplasmic retention of DAF‐16/FoxO, involving 3‐phosphoinositide‐dependent kinase‐1 (PDK‐1).

Lee *et al*. showed that adding 2% glucose to bacterial diet of *C. elegans* shortened the lifespan by inhibiting the activity of DAF‐16/FoxO and heat‐shock factor HSF‐1. This effect involves the downregulation of an aquaporin glycerol channel, *aqp‐1*, which is also inhibited by glucose in mammals. Moreover, when 2% glucose was provided to *C. elegans* insulin/IGF‐1 receptor mutants, their lifespan extension was nearly completed repressed (Lee *et al*., 2009). The expression of components of the insulin/IGF‐1 longevity pathway in subsets of cells can affect the rate of aging of the entire organism, implying an active coordination of the aging rates between the different tissues to establish homeostasis. Murphy *et al*. (2007) reveal that in *C. elegans*, this communication between the tissues is mediated by INS‐7 (an insulin‐like peptide), which is regulated by DAF‐16/FoxO activity in the intestine, and that this regulation allows DAF‐16 activity in the intestine to influence DAF‐16 activity in other tissues. But insulin‐like peptides may not be the only signals that act downstream of DAF‐16/FoxO to influence lifespan. In *daf‐16*;* daf‐2* double mutants, the expression of DAF‐16 specifically in one tissue (intestine/adipose tissue) can increase the worm lifespan by 60% (Libina *et al*., 2003). As nonintestinal tissues do not contain DAF‐16/FOXO in these double mutant animals, it must influence other downstream longevity signaling pathways in those tissues. The secreted peptide SCL‐1 is a candidate for such a downstream signal (Ookuma *et al*., 2003).

Several reports indicate that FoxO induces autophagy acting in a non‐cell‐autonomous manner, evidencing that proteostasis is beneficial for longevity. Melendez *et al*. (2003) showed that autophagy is a cellular pathway essential for dauer development and lifespan extension in *C. elegans* (Melendez *et al*., 2003). Using *daf‐2* mutants, the authors demonstrate that *bec‐1*, the *C. elegans* ortholog of mammalian autophagy gene APG6/VPS30/beclin1, is essential for normal dauer morphogenesis and lifespan extension. Dauer formation is associated with increased autophagy and requires autophagy genes APG1, APG7, APG8, and AUT10 (Melendez *et al*., 2003). Accordingly, increased autophagy is essential for lifespan extension in dietary restriction conditions or TOR inhibition in *C. elegans* (Hansen *et al*., 2008). But, whereas DAF‐2 mutants require both autophagy and the transcription factor DAF‐16/FoxO to promote longevity, autophagy takes place in the absence of DAF‐16/FoxO. This may suggest that autophagy provides raw material for new macromolecular synthesis that requires the action of DAF‐16/FoxO, recycling this material into cell‐protective longevity proteins (Hansen *et al*., 2008). In *Salmonella*‐infected worms, inactivation of the autophagy pathway was shown to increase bacterial intracellular replication, reducing animal lifespan, culminating in an apoptotic‐independent death (Jia *et al*., 2009). Mutation of DAF‐2 or overexpression of the DAF‐16/FoxO conferred pathogen resistance, which is precluded with the genetic knockdown of autophagy genes (Jia *et al*., 2009).

Lifespan in *C. elegans* can also be modulated by its reproductive system. The removal of the worm′s germ cells extends its life by 60% (Hsin & Kenyon, 1999; Kenyon, 2010b). Germ line loss stimulates nuclear accumulation of DAF‐16/FoxO and the expression of the transcription elongation/splicing factor homolog TCER‐1 in intestinal cells. Although the precise mechanism of how the information about the reproductive status is transferred to the intestine is unclear, it has been shown that the intestinal adaptor protein KRI‐1 is required to induce DAF‐16/FoxO target gene expression and extend lifespan (Berman & Kenyon, 2006; Ganapathy *et al*., 2010). This pathway is apparently independent of the IIS and, accordingly, in *daf‐2* mutants, the loss of germ line acts additively with the IIS to expand the long lifespan of these mutants. Wang *et al*. (2008) proposed that this lifespan expansion may be related to lipid metabolism, by showing that *C. elegans* germ line stem cells actively promote systemic lipolysis via induction of a specific triglyceride lipase, identified as lipl‐4. As DAF‐16/FoxO upregulates this triglyceride lipase in response to germ line removal, it has been suggested it may be involved in the pathway that allows DAF‐16/FoxO activity in the worm's intestine/adipose tissue to influence *C. elegans* lifespan (Wang *et al*., 2008; Kenyon, 2010a).

### *Drosophila melanogaster*

*Drosophila melanogaster*, or fruit fly, is an invertebrate of the taxonomic order *Diptera*. It has a lifespan of about 30 days and a four‐stage life cycle: egg, larva, pupa, and adult. Since 2001, it is known that the downregulation of IIS could extend lifespan in the fruit fly *D.melanogaster*, establishing the evolutionary conserved role of this pathway in aging (Clancy *et al*., 2001; Tatar *et al*., 2001). Mutation of *Drosophila* InR (insulin‐like receptor) homologous to mammalian insulin receptors yields dwarf females with up to an 85% extension of adult longevity and dwarf males with reduced late age‐specific mortality (Clancy *et al*., 2001; Tatar *et al*., 2001). Conversely, treating these long‐lived InR‐mutated dwarfs with a juvenile hormone analog restores life expectancy toward that of wild‐type controls (Clancy *et al*., 2001; Tatar *et al*., 2001). *Drosophila* gene chico (named after the small size of the correspondent mutants) encodes an insulin receptor substrate that functions in the IIS pathway. Mutation of this gene extends fruit fly median lifespan by up to 48% in homozygotes and 36% in heterozygotes (Clancy *et al*., 2001). Importantly, this lifespan extension was not a result of impaired oogenesis in chico females nor was correlated with increased stress resistance (Clancy *et al*., 2001).

dFoxO, the equivalent of nematode DAF‐16/FoxO and mammalian FOXO3A, has been shown to be a key transcriptional regulator of the insulin pathway that modulates growth and proliferation in *D.melanogaster* (Puig & Mattila, 2011). Without ligand binding at the insulin‐like receptor, dFoxO remains unphosphorylated and translocated to the nucleus, activating expression of factors that retard cell growth and proliferation (Junger *et al*., 2003; Puig *et al*., 2003). On the other hand, insulin treatment leads to dFoxO phosphorylation by dAKT, leading to cytoplasmic retention and inhibition of its transcriptional activity. Mutant dFoxO lacking dAKT phosphorylation sites does not respond to insulin inhibition and is constitutively active in the nucleus (Puig *et al*., 2003). dFoxO activation induces growth arrest and increases the expression of two key players of the dInR/dPI3K/dAKT pathway: the translational regulator d4E‐BP and the dInR itself. Interestingly, targeted expression of dFoxO in fly tissues regulates organ size by specifying cell number with no effect on cell size (Puig *et al*., 2003).

As in the worm, the nervous system has been shown to be implicated in the IIS‐mediated extension of lifespan in *Drosophila* (Fontana *et al*., 2010). *Drosophila* has seven genes encoding insulin‐like peptides (dilps), and ablation of the cells expressing only three of the seven dilps in neuroendocrine cells of the brain is sufficient to increase longevity (Broughton *et al*., 2008). dFoxO has also been shown to regulate *D. melanogaster* aging when activated specifically in the adult pericerebral fat body. This limited activation of dFoxO reduces expression of the peptide dilp‐2 synthesized in neurons and represses the IIS pathway in peripheral fat body. These findings suggest that autonomous and nonautonomous roles of insulin signaling concomitantly contribute to control aging (Hwangbo *et al*., 2004).

Dietary restriction has been shown to affect the expansion of lifespan in flies mediated by both TOR and IIS pathways (Kenyon, 2010b). The fall in nutrients leads to a diminished TOR activity, increasing lifespan by two possible mechanisms: by inhibiting general translation and increasing respiration or by enhancing autophagy (Kenyon, 2010b).

Interestingly, life extension through dietary restriction does not require dFoxO (Giannakou *et al*., 2008). Nevertheless, the overexpression of dFoxO in the adult fat body and gut of *Drosophila* showed an altered response to dietary restriction, behaving as partially dietary restricted. The authors suggest that, although dFoxO is unnecessary to extend lifespan of flies in response to dietary restriction, the presence of active dFoxO modulates the response to dietary restriction (Giannakou *et al*., 2008). This likely occurs by changing the expression of dFoxO‐target genes, indicating that dFoxO may mediate the normal response to dietary restriction (Giannakou *et al*., 2008). Min *et al*. (2008) showed evidence that the diet‐dependent effects of dFoxO overexpression on fly's lifespan are associated with reduction of dilp2.

The stress‐responsive JNK pathway also requires dFoxO to extend lifespan in *Drosophila* (Wang *et al*., 2005). JNK has been shown to antagonize IIS, promoting dFoxO nuclear localization, therefore inducing expression of growth control and stress defense genes. Moreover, the repression of IIS ligands by JNK and dFoxO in neuroendocrine cells systemically downregulates the IIS pathway (Wang *et al*., 2005).

As in *C. elegans*, dFoxO can regulate autophagy in *Drosophila* at an organismal level. Overexpression of dFoxO or the dFoxO‐target 4E‐BP in the muscle reduces protein aggregation in other tissues (brain, adipose tissue, and retina), delaying muscle functional decay and extending lifespan (Demontis & Perrimon, 2010). Moreover, dFoxO/4E‐BP overexpression in muscle decreases feeding behavior and the release of insulin, therefore delaying the age‐related accumulation of protein aggregates in other tissues. These results reveal a non‐cell‐autonomous mechanism mediated by dFoxO/4E‐BP signaling in the coordination of organismal and tissue aging (Demontis & Perrimon, 2010).

### Mammals

The family of mammalian FOXO transcription factors comprises four members. FOXO1, FOXO3, and FOXO4 are highly related, share the same DNA‐binding motifs, present similar patterns of expression, and seem to have overlapping functions (Anderson *et al*., 1998; Biggs *et al*., 2001; Jacobs *et al*., 2003; Obsil & Obsilova, 2011). FOXO6 is mainly expressed in the brain and has been shown to be regulated by distinct mechanisms. Interestingly, knockouts of single Foxo genes in mice present with very distinct outcomes: Foxo1 knockout mice die *in utero* due to defects in vasculature (Furuyama *et al*., 2004; Hosaka *et al*., 2004). Female Foxo3 knockout mice were found to be sterile due to global primordial follicle activation with subsequent oocyte exhaustion (Castrillon *et al*., 2003; Hosaka *et al*., 2004). In addition, depletion of Foxo3 resulted in deficient development of regulatory T cells with consequent organ inflammation by a mechanism also involving Foxo1 (Harada *et al*., 2010; Kerdiles *et al*., 2010). Foxo4 and Foxo6 knockout mice present with only mild phenotypes (Zhu *et al*., 2011; Salih *et al*., 2012). Accordingly, conventional genetic analysis did not reveal an overt tumor‐prone or hematopoietic phenotype for the deficiency of any one of the Foxo family members. As closely related members of gene families may mask individual gene functions in single knockout experiments, conditional alleles for Foxo1, Foxo3, and/or Foxo4 have been generated. Paik *et al*. (2007) showed that triple knockout mice prompted a progressive cancer‐prone condition characterized by thymic lymphomas and hemangiomas which lead to early death. This study established mammalian Foxo proteins as *bona fide* tumor suppressors and confirming the functional redundancy of Foxo family members. However, it still remains to be determined why these animals did not present neoplastic phenotypes in epithelial tissues. Alternative downstream arms of the PI3K/AKT pathway might play a more prominent trumorigenic role in epithelial compartments. Conditional deletion of Foxo1, Foxo3, and Foxo4 in the adult hematopoietic system resulted in a marked context‐dependent increase in ROS in Foxo‐deficient HSC compared with wild‐type HSC that correlated with changes in expression of genes that regulate ROS. The number and the long‐term repopulating activity of HSC were found to be significantly reduced in these animals (Tothova *et al*., 2007). These results highlight the importance of mammalian Foxo proteins for the long‐term regenerative potential of HCS (Tothova & Gilliland, 2007). Ablation of the three Foxo proteins resulted in an increase in oxidative stress in bone and osteoblast apoptosis and a decrease in the number of osteoblasts, the rate of bone formation and bone mass (Ambrogini *et al*., 2010). Moreover, the overexpression of a Foxo3 transgene in mature osteoblasts decreased oxidative stress and osteoblast apoptosis and increased osteoblast number, bone formation rate, and vertebral bone mass. These authors concluded that Foxos provide an oxidative defense mechanism to deal with the aerobic metabolism of osteoblasts, being indispensable for bone mass homeostasis (Ambrogini *et al*., 2010). Numerous research groups have used the same approach to conditionally delete Foxo1, Foxo3, and Foxo4 in different tissues, but the effect in aged knockout mice has not been examined. There is no doubt that in the near future experiments will shed light on this central aspect of FOXO biology. Intriguingly, recent studies show that the life‐extending effect of dietary restriction requires Foxo3 but not Foxo1 in mice (Yamaza *et al*., 2010; Shimokawa *et al*., 2015). The mechanism(s) by which Foxo factors contribute to lifespan remain elusive. As previously mentioned, decreased autophagy has been related to premature aging and age‐related associated disorders (Hara *et al*., 2006; Komatsu *et al*., 2006; Jung *et al*., 2008; Pickford *et al*., 2008; Masiero *et al*., 2009; Lee *et al*., 2010a, 2010b). Foxo factors have been shown to regulate autophagy in mouse muscle, particularly Foxo3, which induces the expression of several autophagy genes and increases autophagosome formation (Mammucari *et al*., 2007; Zhao *et al*., 2007; Webb & Brunet, 2014). Foxo1 and Foxo3 overexpression also promotes mitophagy (degradation of mitochondria by the autophagy–lysosomal pathway) by upregulating the expression of the mouse mitochondrial E3 ubiquitin protein ligase 1 (Lokireddy *et al*., 2012). Remarkably, Foxos have also been implicated in the autophagy and mitophagy of neurons. FOXO3 has been shown to control the accumulation of human α‐synuclein, a protein known to participate in the development of Parkinson's disease (Pino *et al*., 2014). Mild FOXO3 activity protects nigral neurons against the accumulation of human α‐synuclein by promoting its degradation. These results suggest a determinant role for FOXO3 in Parkinson's disease, via neuronal survival in the substantia nigra (Kume *et al*., 2010). Furthermore, FoxO1 and FoxO3 factors have also shown to promote autophagy in rat neonatal cardiomyocytes and primary renal proximal tubular cells (Sengupta *et al*., 2009; Kume *et al*., 2010).

Warr *et al*. (2013) demonstrated that mouse HSCs robustly induce autophagy and identified Foxo3A as critically important for rapid induction of autophagy upon starvation. Interestingly, these authors also showed that old HSCs retain an intact Foxo3A‐driven pro‐autophagy gene program and that autophagy is required to maintain energy homeostasis and promote survival of the HSCs (Warr *et al*., 2013).

Aging is associated with a decreased proteasomal activity, leading to excess of damaged proteins in muscle, liver, and heart (Conconi *et al*., 1996; Petropoulos *et al*., 2000; Bulteau *et al*., 2002; Husom *et al*., 2004). Foxo3 has been shown to be a transcriptional regulator of muscle‐specific E3 ubiquitin ligases, which are major effectors of protein degradation in muscle (Sandri *et al*., 2004, 2006; Stitt *et al*., 2004). But FOXOs can also affect the composition of the proteasome. In particular, FOXO4 has been shown to be required for the expression of the proteasome component PSMD11 in human embryonic stem cells (Vilchez *et al*., 2012).

## Conclusions

An exciting research area on FOXO transcription factors' impacting on longevity has arisen in recent years. Studies have been conducted to address their upstream regulation, their downstream effectors, and respective signaling pathways in various animal models. Consequently, how these FOXO‐mediated programs affect cellular or tissue function and whether there is an effect at an organismal level, has also been scrutinized. Several lines of evidence suggest that FOXOs affect longevity in a pleiotropic fashion, influencing several cell‐regulated activities such as stress resistance, metabolism, cell cycle arrest, and apoptosis.

A myriad of future work can be envisioned at this time. The induction of FOXO‐mediated programs in tissues with distinct metabolic potential such as brain, muscle, or adipose tissue and with different stages of differentiation or metabolic conditions (nutrition, oxidative stress) will enlarge our knowledge of how FOXO factors affect cellular/organismal lifespan. To further comprehend how FOXOs affect longevity, it is of high importance to understand how human FOXO sequence variants (namely FOXO3A) affect protein expression, its structure, or transcriptional activity. In order to see how these variants translate into physiological profiles, future investigations should address how these variants affect the level of FOXO proteins and their downstream effectors in serum. This approach has been used successfully in patients with vitiligo, in which FOXO3A levels were shown to be decreased when compared with the control group (Ozel Turkcu *et al*., 2014).

Compression of morbidity relates to both extended lifespan and delayed onset of age‐related diseases, such as cancer and cardiovascular disorders. The development of molecules targeting aging mechanisms that underlie a number of age‐related diseases is an exciting field that is nowadays in its first steps. It is noteworthy that clinical trials to test lifespan extension in humans would be challenging and require markers that can detect difference in aging rate across a short time frame. But given the potential of FOXO proteins to impact on numerous disorders such as cancer, diabetes, neurodegeneration, or immune system dysfunction, novel therapeutic modalities based on FOXOs will most likely take place in the near future.

## Conflict of interest

None declared.
